# Selected metabolites profiling of *Orthosiphon stamineus* Benth leaves extracts combined with chemometrics analysis and correlation with biological activities

**DOI:** 10.1186/s12906-015-0884-0

**Published:** 2015-10-07

**Authors:** Noor Hafizoh Saidan, Mohd Shahrul Ridzuan Hamil, Abdul Hakeem Memon, Maha Mansour Abdelbari, Mohammad Razak Hamdan, Khamsah Suryati Mohd, Amin Malik Shah Abdul Majid, Zhari Ismail

**Affiliations:** Department of Pharmaceutical Chemistry, School of Pharmaceutical Sciences, Universiti Sains Malaysia, Minden, 11800 Pulau Pinang Malaysia; Centre of Drug Research, School of Pharmaceutical Sciences, Universiti Sains Malaysia, Minden, 11800 Pulau Pinang Malaysia; School of Agriculture and Biotechnology, Faculty of Bioresources and Food Industry, Universiti Sultan Zainal Abidin, Tembila Campus, 22200 Besut, Terengganu Malaysia; Department of Pharmacology, School of Pharmaceutical Sciences, Universiti Sains Malaysia, Minden, 11800 Pulau Pinang Malaysia

**Keywords:** Metabolites, *Orthosiphon stamineus*, Lamiacea, HPLC, FTIR, Chemometrics tools, PCA, HCA

## Abstract

**Background:**

Studies on selected metabolites profiling of *Orthosiphon stamineus* extracts using chromatographic and spectroscopic techniques combined with chemometric tools have not been fully elucidated. Thus present study was performed to profile selected metabolites in *O. stamineus* leaves extracts using HPLC and FTIR combined with chemometric tools and correlated with biological activities.

**Methods:**

Five different extracts were prepared using three methods; maceration, soxhlet and reflux. The extracts were analyzed using UV-Vis, HPLC and FTIR techniques. Analysis of selected primary and secondary metabolites was also evaluated. The antioxidant and cytotoxic activities of the extracts were evaluated. Chemometric tools were employed to classify the extracts based on HPLC analysis and FTIR fingerprints.

**Results:**

The ethanolic extract using maceration characterized high content of phenolics and flavonoids, (rosmarinic acid and eupatorin) with high antioxidant activity. Ethanolic (50 %) and methanolic extracts using soxhlet showed high proteins and glycosaponins. Water extracts using reflux and maceration showed high polysaccharides. Methanolic extract (50 %) using soxhlet and methanolic extract using maceration showed strong cytotoxic effect against MCF7 and HCT116 cell lines, respectively. Antioxidant and cytotoxic activities showed significant correlation with selected primary and secondary metabolites. HPLC fingerprints combined with chemometrics showed the extracts have been clustered based on selected major peaks profile. FTIR fingerprints combined with chemometrics showed that the extracts have been clustered based on protein and polysaccharide contents.

**Conclusion:**

Ten different extracts of *O. stamineus* have showed significant differences in the content of selected primary and secondary metabolites as well as the biological activities. Chemometric tools were able to classify and discriminate the distinctive features of extracts thus can be correlated with the biological activities.

## Background

*Orthosiphon stamineus* (Cat’s whiskers) is a popular medicinal herb in Southeast Asia and currently cultivated in Malaysia. This herb has gained great interests due to the wide range of pharmacological effects including antioxidant activity [1] and anti-angiogenesis [2]. Based on the great potential of this herb, it has been commercialized for pharmaceutical purposes in Malaysia [3]. Previous phytochemical studies reported that *O. stamineus* leaves contain high contents of phenolic compounds including lipophilic flavones, flavonol glycosides, caffeic acid derivatives such as rosmarinic acid, 2,3-dicaffeoyltartaric acid [4] and other compounds such as diterpenes, triterpenes including betulinic, ursolic, oleanolic acids and β-sitosterol [5].

Previous study also showed the occurrence of primary metabolites (proteins, polysaccharides and saponins) in freeze and spray dried methanol extract (50 %) [2]. Sufficient research work has been done on the analysis of *O. stamineus* [1, 4, 6]. A comparative study of selected metabolites profiling in the different extracts using chromatographic and spectroscopic techniques combined with chemometric tools has not been reported. Previously, a sensor technique combined with chemometric tools (PCA, LDA, HCA and SOM) have been reported for the dried leaves of *O. stamineus* [3, 7, 8].

FTIR and HPLC fingerprints of phytochemicals using chromatographic and spectroscopic techniques, may provide valuable information about qualitative and quantitative analysis of medicinal herb in which pattern recognition can be achieved using chemometric tools including PCA and HCA [9]. Therefore, the aim of this study is to profile selected primary and secondary metabolites in different extracts of *O. stamineus* leaves to correlate the profiles with biological activities using HPLC and FTIR combined with chemometric tools (PCA and HCA) for quality control.

## Methods

### Preparation of raw material

*Orthosiphon stamineus* plant was cultivated and propagated under controlled conditions in a joint venture project of USM-UNIMAP at Titi Tinggi, Perlis, Malaysia. Taxonomic authentication was performed by Mr. Shanmugan A/C Vellosamy (Taxonomist). A voucher specimen (no. 11009) was deposited at the Herbarium, School of Biological Sciences, Universiti Sains Malaysia. The leaves were cut, dried in oven at 40 °C until fully dried and were ground to powder. Powdered leaves were kept in tight container at 25 °C [6].

### Chemicals and reagents

Methanol and ethanol (analytical grade), acetonitrile and formic acid (HPLC grade) were purchased from Merck, Petaling Jaya, (Malaysia). 2,2-diphenyl-1-picrylhydrazyl, quercetin, gallic acid, aluminium chloride, bovine serum albumin, copper sulphate, folin-ciocalteu’s reagent, anthrone, sodium carbonate and potassium tartrate were purchased from Sigma-Aldrich, (Germany). The reference compounds rosmarinic acid, 3′-hydroxy-5,6,7,4′-tetramethoxyflavone, sinensetin and eupatorin were purchased from ChromaDex, (USA). Deionised water for HPLC was prepared using ultra pure water purifier system Thermo Scientific, (USA). The reverse phase Acclaim Polar Advantage II C18 column (3 μm, 3 × 150 mm) was purchased from Dionex, Thermo scientific, (USA). 3-(4,5-dimethylthiazol-2-yl)-2,5-diphenyl tetrazolium bromide, potassium chloride, potassium dihydrogen phosphate, dimethyl sulfoxide and sodium chloride were purchased from Sigma-Aldrich, (USA). Disodium hydrogen phosphate was purchased from Fluka, (Switzerland).

### Preparation of extracts

Three types of extraction techniques; soxhlet, maceration and reflux were used. For soxhlet, dried powdered leaves (250 g) was extracted with methanol, ethanol, methanol (50 %) and ethanol (50 %) in triplicate at 50 °C for 48 h. For water extraction, reflux and maceration techniques were used. The ratio of raw material to solvent was 1:10. For maceration, dried powdered leaves (250 g) was extracted with methanol, ethanol, methanol (50 %), ethanol (50 %) and water in triplicate at 25 °C for 72 h. The extracts were concentrated using rotary evaporator at 60 °C.

### UV-Visible spectra and calculation of extraction factor

UV-Vis spectra were recorded at 600–200 nm for ten different extracts using UV-visible spectrophotometer (Perkin Elmer Lambda 45, USA). Briefly, each crude extract (1 mg) was dissolved in 1 mL of respective extraction solvent. The stock solutions were diluted 10 folds to become 100 μg/mL in methanol (50 %) which was also used as blank. The yield of the extraction in different solvents was calculated using extraction factor (EF) of bioactive molecules from each extract, considering the absorption values (A_max_) recorded for each λ_max_, multiplied with the dilution factor (d). The following formula was used for the calculation of EF:

$$ \mathrm{E}\mathrm{F} = \mathrm{A}\left({\uplambda}_{\max}\right) \times \mathrm{d} $$

The results were expressed as mean values of three replicates per extracts [9].

### Determination of selected primary and secondary metabolites

#### Determination of total phenolic contents

The total phenolic contents of different leaves extracts were determined using folin-ciocalteau’s method [10] with slight modification. Briefly, for a total of 1 mL of mixture; 10 μL of each extract (1 mg/mL) was mixed separately in test tube with of folin-ciocalteau’s reagent (50 μL) followed by the addition of distilled water (790 μL). Each mixture was kept at 25 °C for 10 min before adding 20 % Na_2_CO_3_ solution (150 μL). The samples were incubated at 25 °C for 1 h and the absorbance was measured at λ_max_ 765 nm against blank without extract. The same procedure was repeated for the standard solution of gallic acid and the calibration line was constructed using a standard curve range from 0.049–400 μg/mL of gallic acid. Total phenolics content were expressed as mg of gallic acid equivalents/g of dry extract (mg GAE/g).

#### Determination of total flavonoid contents

The total flavonoid contents of different extracts were determined using aluminium chloride colorimetric method previously reported [11] with. Briefly, quercetin (2 mg) was dissolved in methanol (5 mL) and seriel ten folds dilutions were prepared with methanol. For a total of 1 mL solution, 100 μL of each crude extract (2 mg/mL) was mixed separately with 10 % AlCl_3_ (20 μL), 1 M of sodium acetate (20 μl), methanol (300 μL) and water (560 μL). The samples were kept at at 25 °C for 30 min and the absorbance was measured at λ_max_ 415 nm against blank. The standard curve for total flavonoids was obtained using quercetin standard solution from 0.049–400 μg/mL. Total flavonoid contents were expressed as mg of quercetin equivalents/g of dry extract (mg QE/g).

#### Determination of total polysaccharide contents

Total polysaccharide contents in different extracts were measured according to the previous method [12] with slight modification using glucose as standard. Briefly, each crude extract (10 mg) was mixed with 1 mL of warm ethanol (80 %), vortexed and sonicated for 10 min. The mixtures were then centrifuged at 2700 rpm for 10 min and the supernatants were discarded. This washing step was repeated for 3 to 5 times. To the precipitate, 10 mL of HCl (1.1 %) was added and the mixtures were heated in a water bath at 60 °C for 5 min to dissolve the precipitates. At the end, 5 mL of HCl (1.1 %) and distilled water was added. In separate test tube each mixture (100 μL) was pipetted and volume was made up to 1 mL with distilled water. A standard curve was obtained using glucose as follows; stock solution of glucose (1 mg/mL) was prepared in distilled water. A series of working standards from 20–100 μg/mL was prepared. Anthrone reagent (0.2 %) in H_2_SO_4_ (4 mL) was added separately in each working standard and sample solution. The reaction mixtures were then heated for 8 min using heating water bath at 60 °C and kept at 25 °C to be cooled. The absorbance of mixtures was measured at λ_max_ 630 nm using distilled water as blank. Calibration curve (20–100 μg/mL) of glucose was plotted by measuring the absorbance versus concentration. Total amount of polysaccharide contents in each sample was calculated using calibration curve and result was presented in term of % of total polysaccharides.

#### Determination of total glycosaponin contents

Determination of total glycosaponin contents in each extract was evaluated using the gravimetric [2] method with slight modification. Each crude extract (1 g) was refluxed at 60 °C three times separately using methanol (50 mL) for 30 min. The mixtures were filtered and concentrated to 10 mL using rotary evaporator. The concentrated solution was added dropwise in cold acetone (50 mL) in order to precipitate glycosaponins. Centrifudge was used to fasten precipitation process at 4 °C at 8000 rpm for 10 min. The precipitates were separated from excess acetone. The precipitate was then dried in the oven at 100 °C to constant weight. Means of three experiments were collected and the total saponins estimation was expressed in term of % of total glycosaponins ± SD (*n* = 3).

#### Determination of total protein contents

The estimation of total protein contents for each crude extract was performed as described previously [13] with slight modification. Freshly prepared an analytical reagent was used in the assay where solution A consisted of sodium carbonate (2 % w/v) mixed with sodium hydroxide (0.1 M) and solution B was copper sulphate (1.56 % w/v) added with potassium sodium tartarate (2.37 % w/v). Briefly, solution A (100 mL) was mixed with solution B (2 mL) to prepare the analytical reagent. Bovine serum albumin (BSA) (1 mg/mL) was prepared as standard stock solution. Each crude extract (100 mg) was mixed with distilled water (10 mL) and vortexed shortly. Each mixture was centrifuged for 10 min at 2700 rpm before supernatant (0.1 mL) was withdrawn and transferred to separate test tube and made up to final volume with distilled water (1 mL). Then, the analytical reagent (3 mL) was added in each test tube and mixed well; the reaction mixture was incubated for 10 min at 25 °C. Following the incubation, the folin-ciocalteu’s reagent (200 μL) was added and each mixture was further incubated at 25 °C for another 30 min. The absorbance of each extract was determined at λ_max_ 600 nm againts a blank having all the reagents except the crude extract. BSA (50–250 μg/mL) was used as a standard to obtain calibration curve. The estimation of total protein contents of each crudeextract was calculated. Means of three experiments were collected and the total protein estimation was expressed as % of total protein ± SD (*n* = 3).

### Antioxidant assays

#### DPPH radical-scavenging activity assay

DPPH radical scavenging activity of each crude extract was determined by previous method [14] with some modification. Briefly, each crude extract (1 mg/mL) was dissolved in methanol (50 %). Each sample (100 μL) was added to 100 μL methanolic DPPH (200 μmol/L) and incubated at 25 °C for 30 min in the dark. Ascorbic acid, quercetin and rosmarinic acid (0.39–100 ug/mL) were used as reference standards and the calibration curves were obtained. The amount of remaining DPPH was determined at λ_max_ 517 nm. The results were expressed as % inhibition activity.

### Feric reducing antioxidant power (FRAP) assay

Total antioxidant activity by FRAP assay for each crude extract was determined using previous method [15] with slight modification. Briefly, the stock solution of acetate buffer; (300 mM, pH 3.6), 2,4,6-tris (2-pyridyl)-S-triazine (TPTZ) solution, (10 mM TPTZ in 40 mM/L HCl) and aqueos ferric chloride (20 mM) solution was prepared. From these stock solutions, acetate buffer (25 mL) was mixed with TPTZ (2.5 mL) and FeCl_3_:6H_2_O (2.5 mL) to make a fresh working solution. Each crude extract (1 mg/mL) was dissolved separately in methanol and labelled as respective stock solutions. From each crude extract stock solution (200 uL) was added in 1.5 mL of freshly prepared FRAP reagent (working solution), stirred and incubated for 5 min in dark condition at 25 °C. The absorbance for each mixture was measured at λ_max_ 593 nm using FRAP working solution as blank. A calibration curve of ascorbic acid (0.195-50 μg/mL) was used as reference. The relative activity of each crude extract was also compared with rosmarinic acid beacause this compound is abundant in each crude extract. The result (*n* = 3) was expressed as mg of ascorbic acid or rosmarinic acid equivalents/g of dry extract (mg AAE or RA/g).

### Cytotoxic study

Cytotoxic study of each crude extract was determined using two cancer cell lines including breast cancer (MCF7) and colon cancer (HCT116) cell lines according to the previous methods [16] with minor modification. The assay plates were observed using micro-plate reader (Hitachi U-2000, Japan) at λ_max_ 570 nm absorbance and DMSO (1 %) was used as negative control.

### Instrumentation and chromatographic condition

The chromatographic conditions were already validated by [6]. Briefly, using HPLC system (Agilent 1260 Infinity, USA), the chromatographic analysis was carried out using a reverse phase C18 column (Aclaim Polar Advantage II, USA). The diode array detector (DAD) was used at 320 nm at 40 °C. The gradient elution was used with the mobile phase A (0.1 % formic acid) and B (acetonitrile) with flowrate (1 mL/min), injection volume (5 μL) with 18 min run time.

### Preparation of standard solutions

The marker compounds including rosmarinic acid (RA), 3′-hydroxy-5,6,7,4′-tetramethoxyflavone (TMF), sinensetin (SIN) and eupatorin (EUP) were used to quantify and standardized various crude extracts of *O. stamineus* leaves. Briefly, the stock solution of each standard (1 mg/mL) was prepared in methanol (50 %). The working solution (0.48–250 μg/mL) of each standard were prepared from stock solution for obtaining the calibration curve.

### Preparation of sample solutions for quantification

The quantification of selected four marker compounds was performed using validated HPLC method [6]. Briefly, each crude extract (10 mg) was dissolved in 2 mL of methanol (50 %), sonicated for 10 min and filtered with 0.45 μm membrane filters and analysed by HPLC technique.

### Attenuated Total Reflection Fourier Transform Infrared (ATR-FTIR) spectroscopy

The Fourier transform infrared spectra (FTIR) of each crude extract was recorded in the region from 4000 to 600 cm^−1^ and 16 scans were accumulated for each spectra using attenuated total reflection (ATR) device (Nicolet iS10, Thermo Scientific, USA). The spectral data was processed using Omnic software (Thermo Scientific, USA). The IR measurement was made at a resolution of 4 cm^−1^. The ATR diamond was carefully cleaned with ethanol between measurements and dried before applying new sample.

### Exploratory data

The correlation coefficients between antioxidant and cytotoxicity assays with selected metabolites profile including total phenolic, flavonoid, polysaccharide, protein and glycosaponin contents were calculated using SPSS Statistics 17.0. All data were represented as mean ± SD (*n* = 3).

### Data processing for chemometric analysis

For HPLC, all chromatograms were corrected for baseline and retention time shifted. The peak area over weight (PA/W) of each peak was calculated using Microsoft excel before transfer to PCA. For FTIR data, the baselines of spectra were corrected using Omnic software (Thermo Scientific, USA). In this study, natural clusters of HPLC and FTIR data was analyzed by PCA using The Unscrambler X (CAMO, Trondheim, Norway) software. Hierarchical clustering analysis (HCA) classification was employed on PCA data to discriminate the metabolites of each crude extract.

## Results and discussion

### UV-Vis fingerprints and Extraction factor (EF)

The selection of extraction technique plays an important role for fingerprint analysis of *O. stamineus* extracts. Three different techniques of extraction were performed in present study, namely soxhlet, maceration at 25 °C and reflux. Five different solvents have been chosen including methanol, methanol (50 %), ethanol, ethanol (50 %) and water. From this study, comparative UV-Vis profile of each crude extract was recorded in order to have an integrated image of the differences between solvent type and concentration of bioactive constituents extracted.

The extraction factor (EF) mean values for phenolic acid derivatives at 270–290 nm and flavonoids between 317–340 nm for each crude extract were calculated and presented in Table 1. From the result it has been observed that EF in ethanol extract using maceration technique produced highest concentration of phenolic acid and flavonoid derivatives as compared to other crude extracts while water extract using maceration showed the lowest concentration. This result revealed that solvent and extraction techniques play an important role in extracting the phenolic compounds especially phenolic acid and flavonoid derivatives which also contributed their role in therapeutic effects [17].

**Table 1:** The absorption maxima (λ_max_) in different extracts of *O. stamineus* leaves from UV-Vis and the value of extraction factor (EF)

Extracts	wavelength (λ_max_)	Extraction Factor (EF) ± SD
* meoh-s	329	136.29 ± 0.10
	286	122.85 ± 0.56
* mw-s	328	142.49 ± 0.05
	283	158.25 ± 0.07
* etoh-s	329	107.29 ± 0.02
	283	119.66 ± 0.16
* ew-s	331	108.61 ± 0.01
	284	110.79 ± 0.08
* water-r	329	135.38 ± 0.04
	284	136.77 ± 0.02
* meoh-m	331	125.02 ± 0.02
	285	113.96 ± 0.01
* mw-m	328	161.88 ± 0.01
	283	186.98 ± 0.05
* etoh-m	332	264.83 ± 0.02
	286	228.31 ± 0.05
* ew-m	329	167.21 ± 0.24
	286	135.01 ± 0.05
* water-m	279	104.17 ± 0.01

*meoh-s = methanol soxhlet; mw-s = methanol:water (1:1) soxhlet; etoh-s = ethanol soxhlet; ew-s = ethanol:water (1:1) soxhlet; water-r = water reflux; meoh-m = methanol maceration; mw-m = methanol:water (1:1) maceration; etoh-m = ethanol maceration; ew-m = ethanol:water (1:1) maceration; water-m = water maceration

### Estimation of total primary and secondary metabolites

Quantitative analysis of selected primary and secondary metabolites including flavonoids, phenolics, polysaccharides, proteins and glycosaponins was carried out for ten different crude extracts (Table 2). It was observed that different extraction techniques gave different amount of selected metabolites. The phenolic contents (TPC) were ranged from 108.2 ± 0.9–467.1 ± 1.5 mg/g GAE where macerated ethanolic extract showed the maximum TPC, in contrast with macerated water extract which shown the lowest TPC. This data is in agreement with UV-Vis profile as mention earlier. The variation in TPC values might due to the polarity of the solvents used to extract polyphenols from plant materials. The total flavonoid content (TFC) for different extracts ranged from 69.0 ± 0.1–174.3 ± 0.2 mg/g QE. The result showed that, macerated ethanolic extract consisted the highest total flavonoid, phenolic contents and antioxidant activity. For total protein analysis (TPA), Lowry’s method was used to estimate TPA in different crude extracts of *O. stamineus* leaves. The assay is a colorimetric assay based on cupric ions and follin-ciocalteau’s reagent for phenolics [13].

**Table 2:** Determination of selected primary and secondary metabolites, antioxidant activities and anti-prolferative activities from different extracts of *O. stamineus* leaves

* A	* 1	* 2	* 3	* 4	* 5	* 6	* 7	* 8	
								* 8a	* 8b
* A1	358.2 ± 3.9	158.6 ± 0.1	29.9 ± 0.0	30.4 ± 0.6	0.4 ± 0.0	90.3 ± 0.8	47.7 ± 0.1	51.5 ± 4.0	39.5 ± 3.3
* A2	383.4 ± 1.2	148.6 ± 0.3	31.2 ± 0.2	26.2 ± 0.2	13.5 ± 0.0	86.0 ± 0.3	49.0 ± 0.2	54.3 ± 2.4	36.4 ± 4.1
* A3	289.3 ± 1.0	112.2 ± 0.4	10.9 ± 0.3	18.3 ± 0.2	20.9 ± 0.1	84.4 ± 0.3	31.0 ± 0.1	48.7 ± 0.1	26.3 ± 0.6
* A4	339.6 ± 1.3	148.0 ± 0.2	38.9 ± 0.3	27.5 ± 0.6	14.0 ± 0.0	87.4 ± 0.2	36.4 ± 0.3	46.6 ± 3.3	29.3 ± 3.5
* A5	246.2 ± 2.0	74.9 ± 0.1	22.0 ± 0.2	20.0 ± 0.2	23.7 ± 0.0	74.5 ± 0.2	43.8 ± 0.2	20.2 ± 1.3	32.8 ± 1.9
* A6	296.7 ± 1.0	110.1 ± 0.2	16.3 ± 0.2	21.9 ± 0.8	3.6 ± 0.1	88.7 ± 0.1	40.3 ± 0.1	49.5 ± 1.5	43.7 ± 1.2
* A7	308.5 ± 1.0	94.2 ± 0.1	21.8 ± 0.2	19.1 ± 0.1	0.65 ± 0.01	88.5 ± 0.2	45.7 ± 0.2	44.1 ± 3.3	36.7 ± 1.3
* A8	467.1 ± 1.5	174.3 ± 0.2	11.6 ± 1.4	19.6 ± 0.1	21.7 ± 0.1	94.2 ± 0.2	48.7 ± 0.3	54.2 ± 1.8	24.6 ± 1.9
* A9	251.2 ± 3.6	113.1 ± 0.2	10.1 ± 0.2	16.7 ± 0.1	1.01 ± 0.0	73.2 ± 0.1	21.4 ± 0.4	44.0 ± 3.3	31.3 ± 1.7
* A10	108.2 ± 0.9	69.0 ± 0.1	23.8 ± 0.1	4.0 ± 0.1	25.5 ± 0.1	50.3 ± 0.2	20.0 ± 0.4	8.5 ± 0.6	40.4 ± 3.0

*A = Extracts; A1 = meoh-s; A2 = mw-s; A3 = etoh-s; A4 = ew-s; A5 = water-r; A6 = meoh-m; A7 = mw-m; A8 = etoh-m; A9 = ew-m; A10 = water-m; 1 = TPC (mg/g GAE) ± SD, 2 = TFC (mg/g QE) ± SD, 3 = Total protein (%) ± SD, 4 = Total saponins (%), 5 = Total saccharide (%) ± SD, 6 = DPPH (% inhibition) ± SD, 7 = FRAP (mg/g AAE) ± SD, 8 = MTT (% inhibition) ± SD, 8a = MCF7 ± SD, and 8b = HCT116 ± SD

TPA for different crude extracts was ranged from 10.1 ± 0.2–38.9 ± 0.3 % w/w of dry extract where ethanolic (50 %) extract using soxhlet showed the highest contents of TPA while ethanolic (50 %) macerated extract showed lowest TPA contents. Methanol extract using soxhlet showed the highest content of total glycosaponin with 30.4 ± 0.6 % w/w of dry extract while water extract using maceration showed the lowest contents of total glycosaponins with 4.0 ± 0.1 % w/w of dry crude extract. In contrast, water extracts using reflux and maceration showed high contents of total polysaccharides with 23.7 ± 0.0 % and 25.5 ± 0.1 % w/w of dry crude extracts, respectively. The result showed that the polysaccharide contents in this plant are water soluble and the extraction technique with controlled temperature preserved the primary metabolites in these extracts. Among selected five phyto-constituents, total polysaccharide contents were found in least amount in all samples except in water crude extracts. The correlation among selected five phyto-constituents was calculated using Pearson’s correlation as shown in Table 3. All selected phyto-constituents are positively correlated except the polysaccharides which are negatively correlated to the rest of the phyto-constituents indicating a decrease in its value along the increase in the other phyto-constituents.

**Table 3:** Correlation among the selected phytoconstituents and activities observed in different extracts of *O. stamineus* leaves

Variables	Phenolic	Flavonoids	Protein	Saponins	Saccharides	DPPH	FRAP	MCF7	HCT
Phenolic	1.00	0.89^**^	0.07	0.73^*^	−0.14	0.90**	0.78^**^	0.85**	−0.44
Flavonoids	0.89^**^	1.00	0.20	0.69^*^	−0.15	0.73*	0.71^*^	0.81**	−0.35
Protein	0.07	0.20	1.00	0.46	−0.07	0.36	0.28	−0.05	0.29
Saponins	0.73^*^	0.69^*^	0.46	1.00	−0.44	0.87^**^	0.70^*^	0.73^*^	−0.06
Sacharides	−0.14	−0.15	−0.07	−0.44	1.00	−0.46	−0.12	−0.76^*^	0.45
DPPH	0.90^**^	0.73^*^	0.36	0.87^**^	−0.46	1.00	0.76^*^	0.89^**^	−0.26
FRAP	0.78^**^	0.71*	0.28	0.70^*^	−0.12	0.76^*^	1.00	0.52	0.01
MCF7	0.85^**^	0.81^**^	−0.05	0.73^*^	−0.76*	0.89^**^	0.52	1.00	−0.25
HCT116	−0.44	−0.35	0.29	−0.06	−0.45	−0.26	0.01	−0.25	1.00

Significant levels were showed as *= *p *<0.05; **= *p* <0.01

### Antioxidant assay

Antioxidant properties of ten different crude extracts were analyzed using DPPH and FRAP assays. The antioxidant activity of different crude extracts is shown in Table 2 where water extracts exhibited low antioxidant activity with both antioxidant methods. This could be due to low contents of total phenolic and flavonoid in these crude extracts.

From the correlation study (Table 3), DPPH showed significant correlation with total phenolics, flavonoids and saponins while FRAP showed significant correlation with total phenolics and saponins. This result also corroborate with previous result where it showed DPPH and FRAP assays have significant correlation with total phenolic contents [15]. Previous study had also reported that apart from phenolic compounds, other compounds such as ursolic, oleanolic and betulinic acids present in *O. stamineus* might also contributed to antioxidant activity [1]. This data also indicated that the antioxidant activitiy of different crude extracts varied due to polarities of different solvents.

### Cytotoxic activity

This study evaluated the cytotoxic activity of ten different crude extracts using two cancer cell lines including MCF7 and HCT116 (Table 2). From this result, the activity varied between different crude extracts using different extraction techniques. Various extracts (100 μg/mL) possessed cytotoxic activity on MCF7 and HCT116 ranged from 8.5 ± 0.6–54.26 ± 2.4 % and 24.6 ± 1.9 %–43.7 ± 1.2 % inhibition, respectively. Methanolic extract (50 %) using soxhlet showed the strongest cytotoxic effect against MCF7 with 54.3 ± 2.4 % inhibition while water exract using maceration showed the weakest activity with 8.5 ± 0.6 % inhibition.

From Table 3, this result indicated that the cytotoxic activity is related to the antioxidant properties of crude extracts. MCF7 showed significant correlation with DPPH (0.89, *p* <0.01) while HCT116 showed negative correlation with DPPH (−0.26, *p* >0.05) and non-significant withh FRAP assay (0.01, *p* >0.05). MCF7 also showed significant correlation with total saponins (0.73, *p* <0.05 ), phenolics (0.85, *p* <0.01) and flavonoid contents (0.81, *p* <0.01) and negatively correlated with total polysaccharides (−0.76, *p* <0.05). Previous study reported that antioxidant activity contributed significantly to the cytotoxic activity of *O. stamineus* extract [18].

### HPLC profile and quantitative analysis

**Fig. 1:**
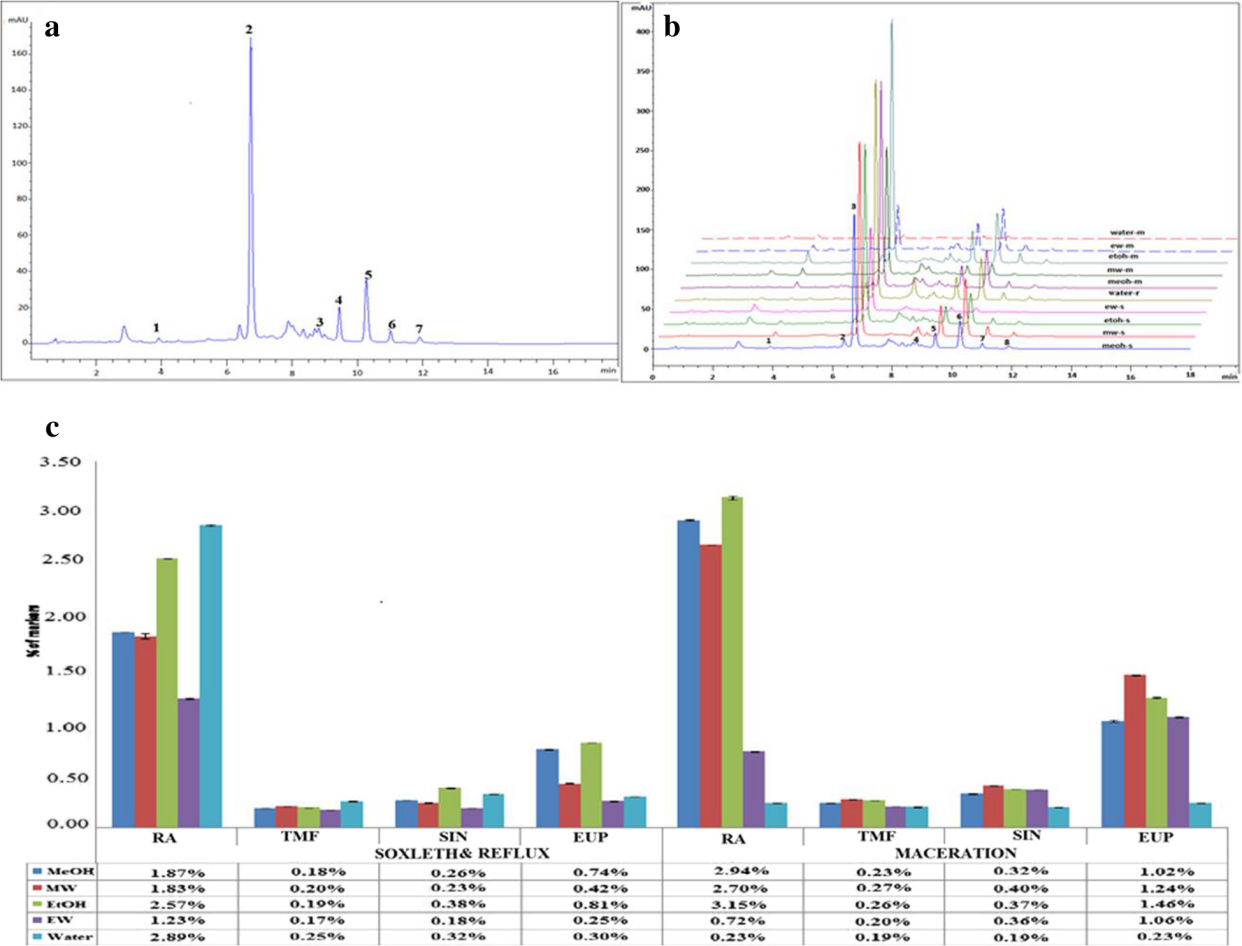
Common pattern of HPLC fingerprints of extracts of *O. stamineus* leaves (**a**) overlapped HPLC chromatogram for ten different extracts (**b**) and quantification of four marker compounds in different extracts of *O. stamineus* leaves (*n* = 3) (**c**)

HPLC analysis was carried out to evaluate the quality of *O. stamineus* extracts based on selected marker compounds (RA, TMF, SIN and EUP). In order to standardize the HPLC profile, crude extracts were analyzed using an established method [6]. Figure 1 shows a typical HPLC fingerprints and the overlapped chromatograms of ten different crude extracts using maceration, soxhlet and reflux techniques. Seven common peaks were selected as characteristic peaks. Out of seven common peaks, only four peaks were quantified based on the comparison of retention time with pure standards and DAD profile. Accordingly, compounds labelled 2, 3, 4 and 5 were identified as RA, TMF, SIN and EUP, respectively.

From Fig. 1c, among two techniques, ethanolic extract from maceration showed high contents of RA and EUP with 3.15 % w/w and 1.46 % w/w, respectively. While water extract (reflux) showed high contents of RA with 2.89 % w/w followed by ethanolic and methanolic extracts (soxhlet) with 2.57 % and 1.87 %, respectively. This result shows a good agreement with UV-Vis profile as mention earlier where ethanolic extract from maceration showed high absorption at 270–290 nm (phenolic acids) and 317–340 nm (flavonoids). The values of TMF and SIN were in the range of 0.17–0.27 % and 0.18 %–0.40 % w/w, respectively. The result also showed that the contents of each marker compounds in different crude extracts were significantly different.

### Chemometrics analysis on HPLC dataset

In order to evaluate the differences of these characteristic peaks, PCA was performed on the basis of the contents of quantified compounds (RA, TMF, SIN and EUP) and PA/W values of remaining peaks in HPLC fingerprints using multivariate statistical (The Unscrambler X CAMO, Trondheim, Norway) software. Data was exported to Microsoft Excel to form a two dimensional matrix (ten samples versus seven variables) which was then exported to statistical (The Unscrambler X, CAMO, Trondheim, Norway) software for PCA and HCA anaysis.

**Fig. 2:**
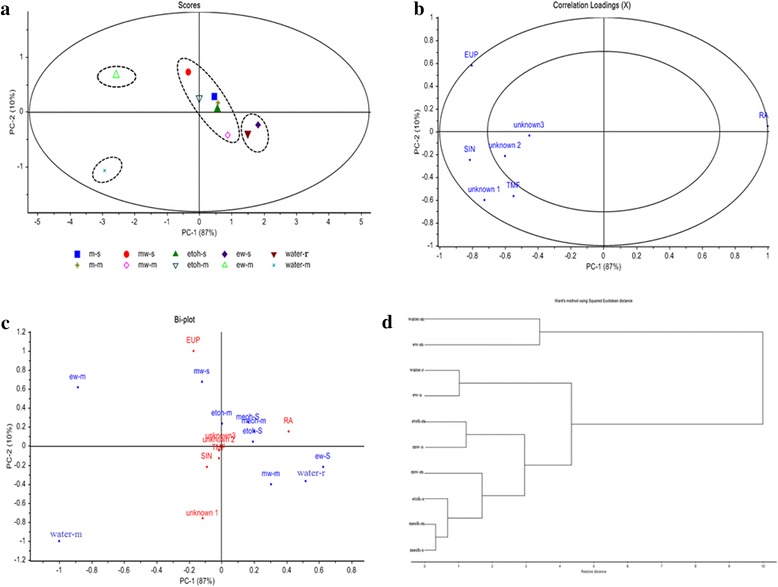
Principle component analysis of different extracts of *O. stamineus* leaves; **a** scores plot, **b** correlation loading, **c** Bi-plot and **d** HCA dendogram based on the PA/W values of seven common peaks

The scores plot obtained from PCA is shown in Fig. 2a. It is shown that the extracts mainly distributed into four groups; group I–IV. Two components, PC-1 and PC-2 showed accounted of 97 % of total variance. From the scores plot, group I and II were in the positive PC-1 axis while group III and IV were in the negative PC-1 axis. Based on the result, those samples with similar chemical profiles are grouped near to each other while larger distance was observed in samples with different chemical profile. To find the variables that contributed to the significant differences between different extracts, the correlation loading plots of PC-1 and PC-2 were generated (Fig. 2b). From correlation loading plots, the PC-1 loading plot indicated that peak 2 (RA) contributed positively to the positions of extracts in group I and II, whereas peak 5 (EUP) contributed in the position of ethanolic extract (50 %) using maceration in group III at negative PC-1 while water extract using maceration (group IV) was influenced by peak 1 (unknown 1). From Fig. 2b, it also showed that peak 6 (unknown 2) and peak 7 (unknown 3) are located in the inner circle, means they did not influenced the discrimination of ten different crude extracts.

Further, to confirm either peak 3 (TMF) or peak 4 (SIN) was contributing to the discrimination of the crude extracts, the Bi-plot was generated and showed (Fig. 2c). From this, it showed that peak 3 (TMF), peak 4 (SIN), peak 6 (unknown 2) and peak 7 (unknown 3) were closed to the centre of origin. It indicates that they did not have enough structural variations to show discriminating factor for the extracts. In this study, group I containing of ethanolic extract (50 %) using soxhlet has the highest PA/W values of peak 2 (RA) followed by water extract using reflux. From group II, ethanolic extract using maceration has the highest contents of peak 2 (RA) followed by methanolic extract using soxhlet and this group has second highest of PA/W values of peak 2 (RA) after group I. From negative PC-1, ethanolic extract (50 %) using maceration has the highest PA/W values of peak 5 (EUP) and negative correlation with peak 2 (RA) while water extract using maceration has the highest PA/W values of peak 1 (unknown 1) and negative correlation with peak 2 (RA).

According to PC-2 scores, methanolic extract (50 %) using soxhlet has second highest of PA/W values of peak 5 (EUP) and peak 2 (RA) and making it’s location on the top in scores plot. Taking together, PCA analysis be able to differentiate and classify the extracts based on the content of seven compounds. Peak 2 (RA) appears in high concentration in all crude extracts and from the loading plot it influences mostly on the separation among different group of samples. Thus, RA also can be used as chemical marker together with SIN in HPLC for quality control of *O. stamineus* extracts in the future. Currently, only SIN has been used as a marker compound for identification and authentication of *O. stamineus* extracts.

### Hierarchical clustering analysis (HCA) for HPLC fingerprinting

The HPLC fingerprint pattern from ten different crude extracts are almost similar. It is difficult to identify the similarity or differences between the extracts. So, in order to assess this tendency, hierarchical cluster analysis was employed. The analysis was performed on the PCA data of values of seven characteristic peaks found in different crude extracts of *O. stamineus* using (The Unscrambler X, CAMO, Trondheim, Norway) software.

The Ward’s hierarchical clustering method with Euclidean distance was used in this study. The dendogram of HCA are shown in Fig. 2d which shows that the extracts were divided into two main clusters; I and II, which further divided into four subgroups; A-D. This cluster was in a good agreement with PCA pattern as discussed earlier. The samples with similar chemical profiles were clustered into the same subgroups. Group I and II were clustered as in Fig. 2d due to the major influenced by peak 2 (RA). Group I containing ethanolic extract (50 %) using maceration was less in PA/W values of peak 2 (RA) but high in PA/W of peak 5 (EUP) and water extract using maceration was also less in peak 2 (RA) but high in peak 1 (unknown 1), so the relative distance between these two extracts is far from each other. Group II containing other extracts were highly in PA/W values of peak 2 (RA) and less PA/W values of peak 1 (unknown 1) and peak 5 (EUP). It was found that different solvents were used in the extraction gave different chemical profiles and clustered separately. Extracts with similar PA/W values were classified in the same group and major peaks (RA) play an important role than small peaks for classification and discrimination of extracts.

### FTIR fingerprints for different extracts of *O. stamineus*

**Fig. 3:**
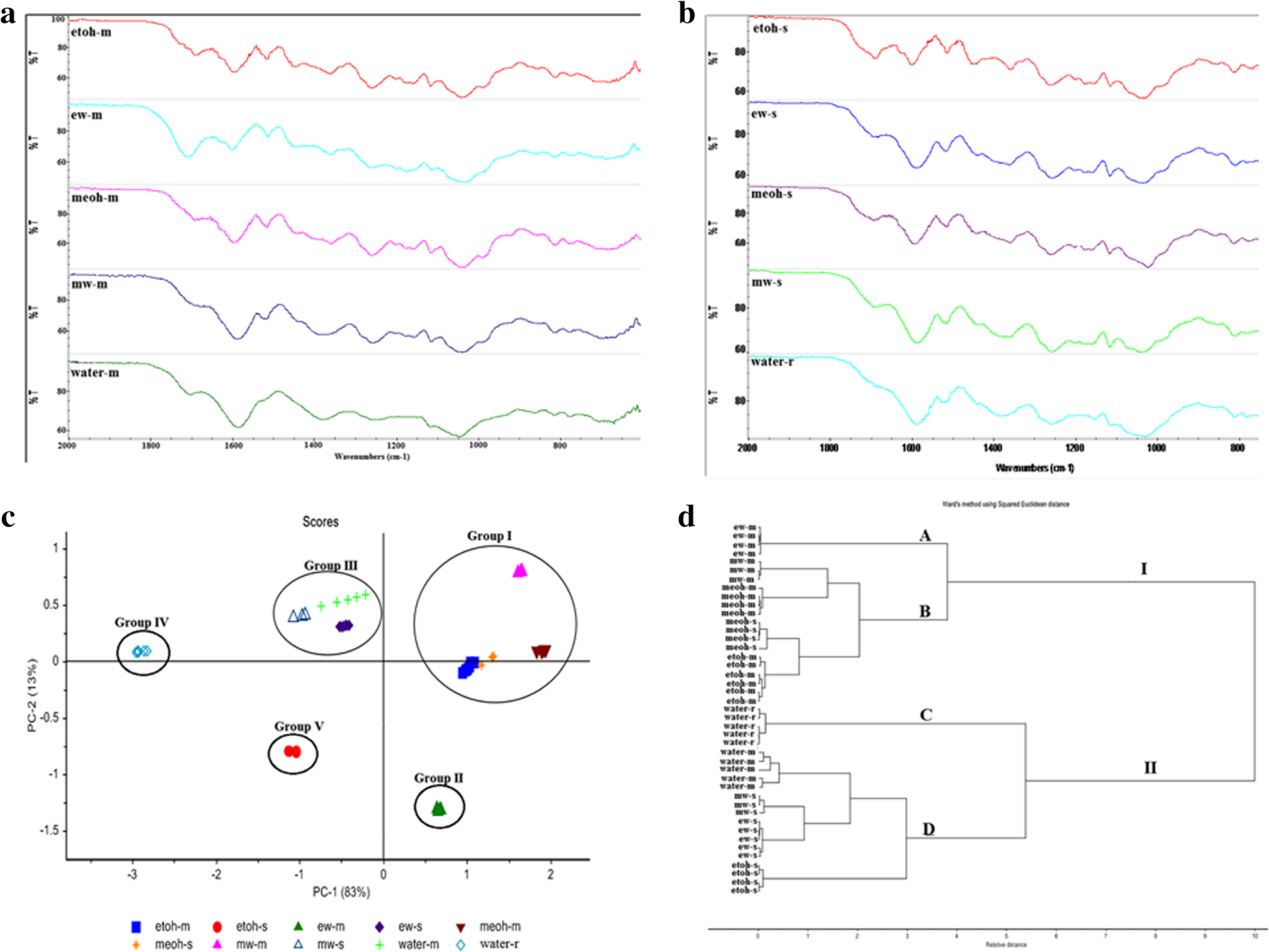
FTIR spectra of different extracts of *O. stamineus* leaves using; **a** maceration **b** soxleth and reflux (water) with principal component analysis **c** scores plot and **d** HCA dendogram

In this study, FTIR spectra of each crude extract was recorded in IR region from 4000–600 cm^−1^ with 16 scans. The FTIR spectra was used to identify the functional groups in ten different crude extracts. Figure 3a and b present the FTIR spectra of ten different crude extracts extracted from three different techniques; maceration, soxhlet and reflux, respectively. In all crude extracts, the broad peak at 3550–3200 cm^−1^ are due to the intermolecular of hydrogen bonding. The vibration area of 2900–2800 cm^−1^ are assigned to the asymmetric and symmetric stretching vibration of methylene groups (CH_2_ and CH_3_), methoxy derivatives, C-H (aldehydes) including *cis* double bonds [9]. No peak at these region was observed for water extracts.

From Fig. 3a and b, the main differences between the extracts were in the region between 1800–800 cm^−1^ known as fingerprint region [19] which can give more information on the mixture of primary and secondary metabolites. The peak at 1760–1600 cm^−1^ are assigned as N-H bending vibration for amino acid [9], C = O stretching in carbonyl compounds which may be characterized by the presence of high contents of terpenoid and flavonoid in the complex mixtures of extracts. With respect to the phenolic compounds, the absorption peak at the region 1650–1450 cm^−1^ is due to aromatic ring stretching and the peak at the range of 1420–1330 cm^−1^ is due to O-H in plane deformation absorption bands [20]. The most intense peak at ~1047 cm^−1^ appear in all extracts suggested that the extracts were highly in flavonoid contenst or might be due to the compounds containing alcohol groups through the stretching vibrations of = C-O-C, C-C or bending vibration of C-OH bonds [21].

Peak at 1680–1540 cm^−1^ was appeared in all extracts and assigned as proteins region and the typical fingerprint regions of carbohydrate was observed at the region of 1200–900 cm^−1^ [22]. Fig. 3a and b showed the intensity of peaks in protein regions were higher in all extracts. As showed in Table 2, the order of total protein contents in extracts using soxleth: ethanolic (50 %) > methanolic (50 %) > MeOH > Water (reflux) > EtOH. This same pattern was also found in the extracts using maceration technique. Comparing the intensity of this region (1680–1540 cm^−1^) in ethanolic (50 %) extract using soxhlet and ethanolic extract (50 %) using maceration, the intensity was higher in ethanolic extract (50 %) using soxhlet showed that the total protein contents in this extract is higher than ethanolic extract (50 %) using maceration. Meanwhile, the intensity of this region are lower in ethanolic extract using soxhlet and maceration, showed that the total protein contents are low in these extracts. The presence of protein in the extracts was further confirmed by the peak region at 1550–1504 cm^−1^ indicating the presence of N-H bending vibration of primary amines and the presence of strong peak at 1515 cm^−1^ indicated for secondary aromatic amines. The absorption peak at region 1275–1200 cm^−1^ was associated to the assymetrical C-O-C stretching.

### Chemometric analysis on FTIR dataset

The PCA was performed on the FTIR spectral features between the wavenumber region of 1800–800 cm^−1^ which is known as fingerprint region [19] using (The Unscrambler X, CAMO) software. Figure 3a and b, showed that the FTIR fingerprints in all extracts look similar and only intensity of the peaks is different. So, to identify the similarity or diffferences between extracts, the PCA scores plot of ten different extracts were generated (Fig. 3c). The scores plot were generated from comparisons of the two PCs; PC-1 and PC-2 which encounted of 96 % of total variants.

A significant separation was observed in all extracts revealed the differences in chemical composition among them. Therefore, in two principle components, there are able to form a cluster in the two dimensional plot. According to the scores plot, there are five different groups; groups I–V. Group I and II were clustered at positive PC-1 axis. Group I and II containing extracts (maceration) except one extract from soxhlet (MeOH) which is in group I while at negative PC-1 axis, group III–V containing extracts from soxhlet except water extract (maceration) which is in group III. It might be due to the chemical profile of the extracts in FTIR spectra is similar. Those samples with similar chemical profiles are near to each other while the larger the distance showed differences in their chemical profiles.

In order to identify the peaks that contributed to the discrimination of extracts, loading plots of PC-1 and PC-2 was generated and it showed that peak at 1275–1200 cm^−1^ (C-O-C stretching) and 1200–900 cm^−1^ (carbohydrates) were the main variants for positive and negative PC-1 loading while peak at 1760–1600 cm^−1^ (amino acids, terpenoids and flavonoids), 1680−1540 cm^−1^ (proteins) and 1420–1330 cm^−1^ (O-H ) were contributed for clustering both positive and negative PC-2 loading. High intensity of the peak at 1200–900 cm^−1^ corresponding to the carbohydrate region compared to other peak indicating that this peak mostly contributed to the discrimination of the extracts in positive and negative PC-1 axis.

Table 2 shows the result of total polysaccharides in ten different crude extracts. All extracts in group I and II showed a lower contents of total polysaccaride while group at negative PC-1 loading showed higher contents of total polysaccharide. For positive and negative PC-2 axis, there are three peaks that influenced the discrimination of the extracts including the region of proteins (1680–1540 cm^−1^). Extracts in positive PC-2 axis showed high contents of total protein while extracts at negative PC-2 axis showed low contents of protein. From the PCA result, proteins and polysaccharides regions play an important role for the discrimination of the *O. stamineus* extracts in scores plot. Further research in these region is needed in order to understand the discrimination obtained from PCA.

### Hierarchical clustering analysis (HCA) for FTIR fingerprinting

In order to see the similarities and dissimilarities between FTIR fingerprints of different extracts, the cluster analysis was conducted using (The Unscrambler X, CAMO, Trondheim, Norway) software. The Ward’s hierarchical clustering method with Euclidean distance was used in this study. The dendogram of HCA obtained by the PCA on FTIR data are shown Fig. 3d. From these, the extracts were grouped into two major clusters; I and II. Group I was divided into two subgroups; A and B where subgroup A consists of macerated ethanolic extract (50 %) while subgroup B was further divided into two subgroups which consists of macerated methanolic (50 %), methanolic, ethanolic and methanolic extracts.

Group II was further divided into subgroups C and D, where subgroup C consists of water extract using reflux while subgroup D was further divided into two subgroups that consists of macerated water extract, methanolic (50 %), ethanolic (50 %) and ethanolic extracts using soxhlet. This cluster was in a good agreement with PCA pattern as discussed above. The samples with similar chemical profiles were clustered into the same subgroups. Group I and II were clustered as in Fig. 3d due to the major influenced by total polysaccharide and protein contents. Thus, grouping in HCA of different extracts of *O. stamineus* confirmed the clustering in PCA scores where group I was clustered in positive PC-1 axis while group II was clustered in negative PC-1 axis.

## Conclusions

To our best knowledge, studies on selected metabolites profiling of *O. stamineus* leaves extracts using chromatographic and spectroscopic techniques combined with chemometrics tools are scanty. So, this is the first report presenting such an elaborate study. Findings from this study can be employed as a template for quality control either in raw materials, extracts or finish products of *O. stamineus* leaves extracts and would be an important tool for selection of potential extracts for further product development study of *O. stamineus* leaves extracts.

## Abbreviations

DPPH: 2,2-diphenyl-1-picrylhydrazyl
(AlCl_3_): Aluminium chloride
BSA: Bovine serum albumin
HPLC: High performance liquid chromatograpy
FTIR: Fourier transform infrared spectroscopy
UV/Vis: Utra-violet visible spectroscopy
RA: rosmarinic acid
TMF: 3'-hydroxy-5,6,7,4'-tetramethoxyflavone
SIN: sinensetin
EUP: eupatorin
MTT: 3-(4,5-dimethylthiazol-2-yl)-2,5-diphenyl tetrazolium bromide
DMSO: Dimethyl sulfoxide.
rpm: Revolutions per minute
PBS: Phosphate buffer saline
PCA: Principle component analysis
HCA: Hierarchical clustering analysis
Na_2_CO_3_: sodium carbonate
